# Clinical Outcome of Patients with Gastric, Duodenal, or Rectal Neuroendocrine Tumors after Incomplete Endoscopic Resection

**DOI:** 10.3390/jcm13092535

**Published:** 2024-04-25

**Authors:** Elisabetta Dell’Unto, Matteo Marasco, Mirta Mosca, Camilla Gallo, Gianluca Esposito, Maria Rinzivillo, Emanuela Pilozzi, Federica Orrù, Davide Campana, Sara Massironi, Bruno Annibale, Francesco Panzuto

**Affiliations:** 1 Digestive Disease Unit, Sant’Andrea University Hospital, ENETS Center of Excellence or Rome, 00189 Rome, Italy; elisabetta.dellunto@uniroma1.it (E.D.); matteo.marasco@uniroma1.it (M.M.); gianluca.esposito@uniroma1.it (G.E.); mrinzivillo@ospedalesantandrea.it (M.R.); bruno.annibale@uniroma1.it (B.A.); 2 Department of Medical, Surgical Sciences and Translational Medicine, Sapienza University of Rome, 00189 Rome, Italy; 3 PhD School in Translational Medicine and Oncology, Sapienza University of Rome, 00189 Rome, Italy; 4 Department of Experimental, Diagnostic & Specialty Medicine (DIMES), University of Bologna, 40138 Bologna, Italy; mirta.mosca@unibo.it (M.M.); davide.campana@unibo.it (D.C.); 5 Medical Oncology, IRCCS Azienda Ospedaliero, Universitaria of Bologna, 40138 Bologna, Italy; 6 Division of Gastroenterology, Fondazione IRCCS San Gerardo dei Tintori, 20900 Monza, Italy; c.gallo19@campus.unimib.it (C.G.); sara.massironi@irccs-sangerardo.it (S.M.); 7 Department of Clinical and Molecular Medicine, UOC Anatomic Pathology, Sant’Andrea Hospital, Sapienza University of Rome, 00189 Rome, Italy; emanuela.pilozzi@uniroma1.it (E.P.); federica.orru@uniroma1.it (F.O.)

**Keywords:** duodenum, neuroendocrine tumors, rectum, resection margins, stomach, endoscopy

## Abstract

**Objectives:** Our aim was to investigate the clinical outcome of patients with well-differentiated gastric, duodenal, and rectal neuroendocrine tumors after treatment with incomplete endoscopic resection due to the finding of microscopic positive resection margins (R1). **Methods:** This is a retrospective analysis of consecutive patients with type 1 gastric, non-ampullary non-functioning duodenal, or rectal neuroendocrine neoplasms with positive R1 margins after endoscopic resection. The rate of tumor recurrence and progression-free survival were considered to be the study’s main endpoints. Statistical analysis was performed using MedCalc^®^ v.17 software and a *p*-value of <0.05 was considered significant. A Cox proportional-hazard regression was performed to identify risk factors for disease recurrence/progression. **Results:** After evaluating 110 patients, a total of 58 patients were included in the final analysis (15 gastric NENs, 12 duodenal NENs, and 31 rectal NENs). After evidence of endoscopic R1 resection had been gathered, 26 patients (44.8%) underwent an endoscopic/surgical extension of the previous resection. Tumor progression (all local recurrences) occurred in five out of fifty-eight patients (8.6%) with a median PFS of 36 months. There were no tumor-related deaths. G2 grading and the gastric primary tumor site were the only features significantly associated with the risk of recurrence of the disease (HR: 11.97 [95% CI: 1.22–116.99], HR: 12.54 [95% CI: 1.28–122.24], respectively). **Conclusions:** Tumor progression rarely occurs in patients with microscopic positive margin excision (R1) after endoscopic resection and does not seem to affect patients’ clinical outcomes.

## 1. Introduction

Neuroendocrine neoplasms (NENs) are uncommon, heterogeneous malignancies that arise from the diffuse endocrine system and are characterized by relatively indolent behavior. Most frequently, these neoplasms originate from the digestive system, including the pancreas and the gastrointestinal tract [1].

In general, NENs can be classified as follows: well-differentiated G1 neuroendocrine tumors (NETs), with Ki67 ≤ 2% (NETs G1); well-differentiated G2, with Ki67 ranging from 3%–20% (NETs G2); well-differentiated G3, with Ki67 > 20% (NETs G3); and poorly differentiated neuroendocrine carcinomas (NECs), which are high-grade by definition [2].

Recently, the incidence of gastroenteropancreatic NENs (GEP-NENs) has increased, most likely due to the improvement in and widespread use of diagnostic techniques. The increase in incidence has been observed mainly in the stomach and rectum [3,4]. The prognosis of patients with GEP-NENs may be influenced by several factors, including tumor stage, tumor grade (expressed by the Ki67 index), and primary tumor site [5].

Gastric neuroendocrine neoplasms (g-NENs) can be classified into three groups: (I) type 1, which are associated with chronic atrophic gastritis (CAG); (II) type 2, which are associated with Zollinger–Ellison syndrome in multiple endocrine neoplasia type 1; and (III) type 3, which occur sporadically without any underlying gastric pathology. Type 1 g-NENs are the most common and indolent type—usually G1 (or G2 with low Ki67) with a <5% risk of metastasis and an overall 5-year survival of almost 100%. However, they have a significant risk of recurrence during follow-up (up to 40%) [6].

Duodenal neoplasms (d-NENS) can be classified as follows: functioning or non-functioning (according to the presence of hormone hypersecretion); sporadic or familiar (according to the presence of genetic syndrome); ampullary or non-ampullary (depending on the site of origin) [6]. They are typically small, sporadic, and non-functioning, are usually G1 (or G2 with low Ki67), non-ampullary, and incidentally discovered during routine esophagogastroduodenoscopies [7].

Rectal neuroendocrine neoplasms (r-NENs) are also usually small, low-grade tumors (usually G1 or G2 with low Ki67). In most cases, they are detected incidentally during a colonoscopy performed for screening or for symptoms unrelated to the NENs’ presence [8].

Endoscopic management is the first therapeutic choice for well-differentiated type I g-NETs, non-functioning/non-ampullary d-NETs, and r-NETs. However, the tumor size must be amenable to endoscopic resection (the cut-off level is usually 1 cm or 2 cm, depending on the specific tumor site) [6,8].

Nevertheless, endoscopic resection of these neoplasms may be incomplete, with a microscopic positive resection margin (R1) (either lateral, vertical, or both) in a significant proportion of cases [9]. The impact of this histological finding on the clinical outcome of these patients still remains to be established.

This study targets patients with well-differentiated gastric, duodenal, and rectal NETs. In particular, this study aims to investigate their clinical outcome after treatment with incomplete endoscopic resection due to the finding of microscopic positive resection margins (R1).

## 2. Materials and Methods

This is a retrospective analysis of consecutive patients with type 1 g-NETs, non-ampullary non-functioning d-NETs, or r-NETs with positive R1 margins after endoscopic resection, collected between 2010 and 2021.

Patients with sufficient histological data available to assess endoscopic resection margins (both lateral and vertical) and a minimum of 12 months follow-up data were included in the final analysis.

After classifying and staging the tumors according to the WHO classification/ENETS staging system [2,10], tumors were excluded if they had a presence of locoregional or distant disease or did not have well-differentiated morphology.

The following clinicopathologic data were recorded at the center where the tumor was resected and then reported in a unique anonymized database for data analysis: patient’s gender and age at diagnosis, characteristics of the tumor (primary site, tumor dimensions, proliferative index with Ki67 index value, grading, and staging), type of endoscopic resection (forceps, snare, endoscopic mucosal resection [EMR], endoscopic submucosal dissection [ESD]), histological evaluation of microscopic endoscopic margins (R0 or R1; for R1 resections, lateral, vertical, or both types of margin involvement). The endoscopist decided which type of endoscopic resection to perform based on the tumor’s features. In terms of endoscopic size, this was assessed visually by the endoscopist using the size of the open biopsy forceps as a reference measure of 5 mm.

The rate of tumor recurrence and progression-free survival (PFS) were considered the main study endpoints. Progression-free survival was defined as the interval between the primary endoscopic resection and evidence of disease progression (recurrence of the disease at the exact site of the previous endoscopic resection, occurrence of locoregional, or both, or distant metastasis).

Statistical analysis was performed using MedCalc^®^ v.17 software (MedCalc Software, https://www.medcalc.org/, accessed on 15 April 2024). A *p*-value of <0.05 was considered significant. The distribution of continuous variables was reported as the median and range, whereas frequencies and percentages were given for qualitative variables.

A univariate analysis using Cox proportional-hazard regression was performed to identify risk factors for the occurrence of disease recurrence or progression. Multivariate analysis was not performed given the low number of observed events in the population.

The study was performed according to the STROBE guidelines [11], and was discussed and approved by the internal local board at each center. The research was conducted in accordance with the ethical principles established by the Declaration of Helsinki [12] and with local laws and regulations. Informed consent for data collection was obtained from each patient. Formal approval by the Ethics Committee was not needed given the study’s retrospective design.

## 3. Results

**Figure 1:**
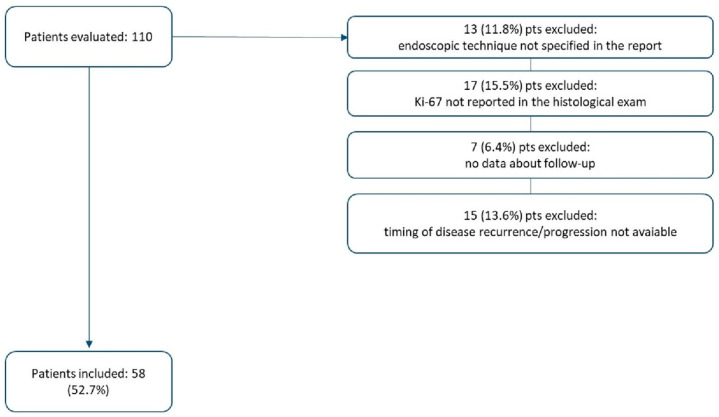
Study population.

A total of 110 patients [M: 57 (51.8%)/F: 53 (48.2%)] were evaluated for inclusion in our study. Of these patients, thirteen patients (11.8%) were excluded because the endoscopic resection technique was not evaluable in the endoscopic report; seventeen patients (15.5%) were excluded because the proliferative index was not expressed in the histological exam (slides for histological revision were not available); seven patients (6.4%) were excluded because follow-up data were not available; and fifteen patients (13.6%) were not included because the timing of the disease recurrence/progression was not specified in the available charts (Figure 1). 

Therefore, the final analysis was performed on 58 patients (52.7%): 33 men (56.9%) and 25 women (43.1%) with a median age of 60.5 years (22–85)] at the time of diagnosis. The characteristics of the patients included in the study are summarized in Table 1.

**Figure 2:**
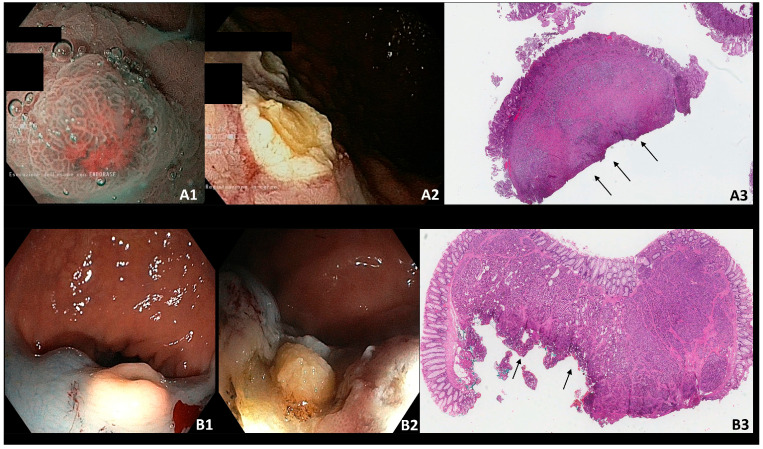
(**A**) Type I g-NEN—incomplete endoscopic resection; (**A1**): type 1 g-NEN in NBI (Narrow Banding Imaging); (**A2**): residual tissue after endoscopic resection; (**A3**): histological slide with positive deep resection margin (arrows). (**B**) R-NEN–incomplete endoscopic resection; (**B1**): r-NEN in white light image; (**B2**): residual tissue after endoscopic resection; (**B3**): histological slide with positive deep resection margin (arrows).

Overall, twenty-four lesions (41.4%) were resected using forceps, five (8.6%) using a cold snare, ten (17.2%) using a diathermic snare, nine (15.6%) by performing an EMR, and ten (17.2%) by performing an ESD. Two cases of ESD resections are shown in Figure 2.

No significant difference was observed depending on the primary tumor site: in fact, the endoscopic resection was performed using forceps/snare or EMR/ESD in 66.7% and 33.3% of the cases in the stomach, respectively; in 75% and 25% of the cases in the duodenum, respectively; and in 64.5% and 35.5% of the cases in the rectum, respectively. After the endoscopic resection, the lateral margins were positive in four out of the fifty-eight patients (6.9%), the deep margin was positive in forty-seven out of the fifty-eight patients (81%), and both the lateral and deep margins were affected in seven out of the fifty-eight patients (12.1%).

After evidence of endoscopic R1 resection had been acquired, twenty-six patients (44.8%) underwent an endoscopic/surgical extension of the previous resection: three (11.6%) with forceps, five (19.2%) with cold/diathermic snare, three (11.6%) using EMR, nine (34.6%) using ESD, one (3.8%) using EFTR (Endoscopic Full-Thickness Resection), and five (19.2%) underwent surgeries (the lymphadenectomy was performed in two cases with negative lymph nodes).

In these twenty-six patients, the tumors were located in the rectum (sixteen patients [61.5%]), in the stomach (eight patients [30.8%]), and in the duodenum (two patients [7.7%]), with a median tumor size of 5 mm (2 mm–10 mm). Twenty-two patients (84.6%) were previously treated using forceps or a cold/diathermic snare, while four patients (15.4%) were previously treated with an EMR or ESD. All except two of the previous resections involved a deep margin, and four of them also had a positive lateral margin.

Of the five patients who underwent surgical extension of the previous resection (transanal endoscopic microsurgery in the rectum, wedge resection in the stomach, or gastroduodenal reresection with Roux-en-Y reconstruction in the duodenum), three patients (60%) had a primary tumor site in the rectum, one (20%) in the duodenum, and one (20%) in the stomach, with a median size of 7 mm (3 mm–10 mm). They were all well-differentiated G1 tumors with a median Ki67 value of 2% (1–2%). In this group of patients, the initial R1 endoscopic resection was performed using forceps in one case (20%), a cold snare polypectomy in one case (20%), a diathermic snare polypectomy in one case (20%), an EMR in one case (20%), and, finally, an ESD in one case (20%).

Among those patients who underwent their first endoscopic enlargement after the initial R1 finding, five patients (19.2%) had R1 margins again. These patients underwent endoscopic active surveillance with no evidence of recurrence during the follow-up.

Overall, two out of the fifty-eight patients received medical therapy with octreotide LAR 30 mg (1 injection every 28 days) with a median therapy duration of 15 months. These two patients were diagnosed with G1 duodenal NETs with a median size of 6.5 mm and involving the deep margin. One was resected using forceps and the other using a cold snare. No endoscopic enlargement was planned for either resection, but no progression or tumor-related deaths were observed during the follow-up.

Tumor progression occurred in five out of the fifty-eight patients (8.6%), after a median interval of time of 36 months from the initial resection. The main features of these patients are summarized in Table 2.

Only two deaths (3.4%) occurred during the follow-up period, after a median interval of 111.5 months from the initial diagnosis; however, neither of these deaths were related to the tumor. Considering the entire study population evaluated for the final analysis (58 patients), the median PFS and OS were 24 months and 32.5 months, respectively. The median length of follow-up was 28.5 months.

According to the univariate analysis, performed using Cox proportional-hazard regressions, G2 grading and the gastric primary tumor site were the only features significantly associated with the risk of recurrence of the disease (HR: 11.97 [95% CI: 1.22–116.99], HR: 12.54 [95% CI: 1.28–122.24], respectively). Conversely, the microscopic margin infiltration and the type of primary resection used (advanced endoscopic resection with EMR or ESD versus resection using forceps or snare) were not significantly associated with disease progression/recurrence (Table 2).

Furthermore, although not statistically significant, the size of the primary tumor showed a trend toward an increased risk of recurrence after endoscopic resection, suggesting that a larger tumor might be correlated with an increased risk of disease progression/recurrence (Table 3).

## 4. Discussion

Endoscopic management is widely accepted for small, well-differentiated NETs arising from the stomach, duodenum, and rectum due to their excellent prognosis and low incidence of metastasis [6,8].

Recent knowledge has led to changes, particularly in the management of small type 1 and type 3 gastric NETs. For the former, a simple endoscopic observation is considered safe provided that the Ki67 is low, meaning that the tumor is of type G1 [6]. Regarding sporadic type 3 gastric NETs under one centimeter, provided the Ki67 is low, endoscopic resection is now a viable option compared to surgical intervention, which was recommended in the past regardless of the tumor size for all sporadic gastric NETs [6,13]

There is still debate in the literature about the best endoscopic technique for resection of gastric, duodenal and rectal NETs. Several studies reported a significant proportion of excisions with positive endoscopic resection margins (R1) [14,15,16], particularly in the duodenum. At this site, advanced resection techniques, such as ESD, are more difficult to apply because of an increased risk of complications (bleeding and perforation) due to the anatomy of the duodenal wall [16].

It is expected that R1 findings might be related to an increased risk of lymph node involvement and local disease recurrence [6,8,17]. However, the real impact of R1 histological findings still needs to be clarified, given the lack of solid scientific data [14,18,19,20,21]. In the clinical setting of r-NETs after incomplete R1 initial removal, endoscopic resection of the visible scar by ESD or EFTR has been advised [22]. The likelihood of neoplasm recurrence during follow-up, even after R1 resection, is relatively low. Furthermore, there is no direct correlation between the risk of R1 resection and the recurrence or progression of the disease during follow-up [17,18,19,20,21,22]. Additionally, the data available in the literature are heterogeneous in terms of the endoscopic technique used, the histological characteristics of the NENs, and the time of follow-up or active surveillance.

The present study suggests that even after incomplete endoscopic resection, disease recurrence occurred in a relatively low proportion of patients (8.6%), irrespective of the type of positive margin (lateral or vertical) and the kind of endoscopic resection technique used. However, positive histological R1 endoscopic resection margins do not seem to have a clear impact on patients’ clinical outcomes [23]. In fact, even in those patients who underwent an enlargement after an initial incomplete excision, which again led to an R1 resection finding, no tumor recurrence or tumor-related deaths occurred during follow-up.

In general, in the present study, no patient developed nodal or distant metastasis even after an R1 resection, and the two observed deaths (3.4%) were not tumor-related. This finding illustrates the natural indolent behavior of this subset of NETs [6,8].

Only the gastric tumor localization was statistically significant (HR: 12.54, *p*-value: 0.029) upon examining potential subgroups that were at a higher risk for tumor recurrence after R1 resection. Although this finding does not have a clear explanation, it is reasonable to assume that tumor recurrence may depend on the continuous ECL-like cell growth stimulation induced by hypergastrinemia occurring in CAG [24]. This hypothesis is supported by the evidence reported in several studies, which have shown how frequent recurrence of type 1 g-NETs is, even in sites other than where the tumor had been completely removed by an endoscopy [25,26,27,28].

Furthermore, G2 grading was also related to an increased risk of tumor recurrence after R1 incomplete resection (HR: 11.97, *p*-value: 0.032). Although expected owing to its ability to influence the clinical outcome of patients with GEP-NENs in general [5,29], the specific role of using grading to predict tumor recurrence after incomplete endoscopic resection is not well established yet.

Based on the present study’s main findings, here are some clinical considerations useful for physicians dealing with endoscopic management of gastric, duodenal, and rectal NETs: first, in general, an R1 finding after endoscopic resection may not have a real clinical impact on patients’ clinical outcomes; second, there does not seem to be a significant advantage of using EMR or ESD instead of snare polypectomy, which is usually considered ineffective for removing NETs due to their subepithelial growth; third, gastric and G2 tumors should be approached with a more interventional approach because of their higher risk of recurrence during follow-up—potentially by a step-up approach until complete R0 resection is achieved.

Unfortunately, even though the present study reports potentially interesting novel findings, it has some limitations that need to be taken into account. For instance, this study included a small number of patients, and also had a retrospective design, which inherently presents as a burden for studies investigating rare diseases, including NETs. The stringent selection criteria applied for including patients in the final analysis likely contributed to the reduced sample size. An additional limitation is represented by the heterogeneity of the population, not only in terms of the primary nature of the tumor, but also due to the type of endoscopic resection performed, and the follow-up methods with which the patients were monitored. Further larger and prospective studies are needed to better understand the real significance of incomplete endoscopic resections in patients with a diagnosis of gastric, duodenal, or rectal NETs. In particular, it is necessary to design collaborative studies involving reference centers with experience in the endoscopic management of these patients, conducting a direct comparison between different endoscopic resection techniques, and stratifying patients by known risk factors, such as tumor size and grading.

In conclusion, the present study reports that microscopic positive margin excision (R1) after endoscopic resection does not seem to affect patients’ clinical outcomes. However, stronger scientific data need to be produced to support this finding.

Based on these considerations, discussion within an NET-dedicated multi-disciplinary team in referral centers, which is widely recognized as a means of improving the care quality in patients with NENs [30], is strongly advised for patients with R1 findings after endoscopic resection. This approach can balance the risk of recurrence during follow-up with the risk of potential adverse events related to unnecessary treatment.

## Figures and Tables

**Table 1:** Overall characteristics of the study population.

Feature	N (%)
Overall n. of patients	58
Gender	M: 33 (56.9)/F: 25 (43.1)
Median age at diagnosis (y)	60.5 [range 22–85]
Primary tumor site	
Stomach	15 (25.9)
Duodenum	12 (20.7)
Rectum	31 (53.4)
Median Ki67	1% [range 1–10%]
Grading	
NET G1	46 (79.3%)
NET G2	12 (20.7%)
Median tumor size	6 mm [range 1–16 mm]
T stage	
T1	45 (77.6)
T1a ^†^	6 (10.3)
T1b ^†^	4 (6.9)
T2 ^‡^	3 (5.2)
Staging	
I	55 (94.8)
II	3 (5.2)

^†^ for rectal tumors: T1a size < 1 cm, T1b size 1−2 cm; ^‡^ for rectal tumors T2 classification is for tumors that invade muscolaris propria (not for size > 2 cm).

**Table 2:** Patients with disease progression after endoscopic resection (n = 5).

Feature	N (%)
Primary tumor site	
Stomach	3 (60)
Rectum	2 (40)
Grading	
G1	2 (40)
G2	3 (60)
Median Ki-67 [range]	3% [1–5%]
Median Size [range]	8 mm [4–16 mm]
Type of first resection	
Forceps/snare	2 (40)
EMR/ESD	3 (60)
Margin involvement	
Deep	4 (80)
Both	1 (20)

**Table 3:** Risk factor for tumor recurrence/progression at univariate analysis.

Variable	Hazard Ratio (HR)	*p* Value
Grading G2	11.97	0.032
Primary gastric NET	12.54	0.029
Resection with forceps/snare vs. EMR/ESD	0.59	0.575
Microscopic involvement of lateral resection margin	0.37	0.387
Primary tumor size *	1.25	0.086

* Continuous variable.

## Data Availability

The data presented in this study are available on request from the corresponding author.
